# Impact of Primary RPE Cells in a Porcine Organotypic Co-Cultivation Model

**DOI:** 10.3390/biom12070990

**Published:** 2022-07-16

**Authors:** Natalie Wagner, Armin Safaei, José Hurst, Pia A. Vogt, H. Burkhard Dick, Stephanie C. Joachim, Sven Schnichels

**Affiliations:** 1 Experimental Eye Research Institute, University Eye Hospital, Ruhr-University Bochum, 44892 Bochum, Germany; natalie.wagner@rub.de (N.W.); armin.safaei@rub.de (A.S.); pia.vogt@rub.de (P.A.V.); burkhard.dick@kk-bochum.de (H.B.D.); 2 Centre for Ophthalmology, University Eye Hospital Tübingen, 72076 Tübingen, Germany; jose.hurst@med.uni-tuebingen.de

**Keywords:** co-cultivation, complement system, cytokines, porcine, retina, retinal pigment epithelium (RPE) cells

## Abstract

The pathological events of age-related macular degeneration are characterized by degenerative processes involving the photoreceptor cells, retinal pigment epithelium (RPE), and the Bruch’s membrane as well as choroidal alterations. To mimic in vivo interactions between photoreceptor cells and RPE cells ex vivo, complex models are required. Hence, the aim of this study was to establish a porcine organotypic co-cultivation model and enlighten the interactions of photoreceptor and RPE cells, with a special emphasis on potential neuroprotective effects. Porcine neuroretina explants were cultured with primary porcine RPE cells (ppRPE) or medium derived from these cells (=conditioned medium). Neuroretina explants cultured alone served as controls. After eight days, RT-qPCR and immunohistology were performed to analyze photoreceptors, synapses, macroglia, microglia, complement factors, and pro-inflammatory cytokines (e.g., *IL1B*, *IL6*, *TNF*) in the neuroretina samples. The presence of ppRPE cells preserved photoreceptors, whereas synaptical density was unaltered. Interestingly, on an immunohistological as well as on an mRNA level, microglia and complement factors were comparable in all groups. Increased *IL6* levels were noted in ppRPE and conditioned medium samples, while *TNF* was only upregulated in the ppRPE group. *IL1B* was elevated in conditioned medium samples. In conclusion, a co-cultivation of ppRPE cells and neuroretina seem to have beneficial effects on the neuroretina, preserving photoreceptors and maintaining synaptic vesicles in vitro. This organotypic co-cultivation model can be used to investigate the complex interactions between the retina and RPE cells, gain further insight into neurodegenerative pathomechanisms occurring in retinal diseases, and evaluate potential therapeutics.

## 1. Introduction

The global median age has increased from 1970 to 2019 by almost 10 years, thereby increasing the risk for neurodegenerative age-related diseases [1]. One of the most common neurodegenerative diseases of the eye, leading to irreversible vision loss, is age-related macular degeneration (AMD) [2]. It is a complex and multifactorial disease associated with several environmental and genetic risk factors, including advanced age, smoking, or cardiovascular disease [3,4]. Generally, AMD is classified into two major forms: a dry (non-exudative) or wet (exudative) type. The dry form is typified by a slower geographic atrophy of the retinal pigment epithelium (RPE), Bruch’s membrane, and choroid, leading to a secondary loss of beneath-lying photoreceptors within the macula. The wet form is characterized by choroidal neovascularization up to edema within the subretinal space and vascular leakage due to defects in Bruch’s membrane and in advanced stages, causing severe damage to the photoreceptor cells [5]. Nevertheless, the exact underlying triggers and pathomechanisms of AMD are not yet known.

An impaired function of the RPE can cause a variety of different retinopathies besides AMD, such as retinitis pigmentosa (RP) and Stargardt disease (SD). RP encompasses a group of hereditary disorders of the RPE and the photoreceptor cells. The clinical symptoms of these diseases are typical night blindness and a progressive constriction of the visual field [6]. SD is the most common autosomal recessive inherited macular dystrophy in adults and children. Commonly, the onset of SD is in childhood, with peaks in early adulthood and in later adulthood. Patients show bilateral central visual loss caused by characteristic macular atrophy and yellow-white dots at the posterior pole of the RPE [7]. Because of its wide clinical spectrum and similarities in late onset to other macular dystrophies, SD is hard to be diagnosed and distinguish from AMD [8]. Both RP and SD are degenerative retinal diseases, and their pathogenesis is associated with a RPE dysfunction [9]. Preclinical models of retinitis pigmentosa and macular degeneration revealed restoration of visual function after transplantation of stem-cell-derived RPE cells and photoreceptors [10]. Nevertheless, those models also implied that many underlying mechanisms still need to be better investigated to understand PR loss and, one day, develop therapeutic approaches.

In order to improve this understanding of the exact pathogenesis of mentioned diseases, it is important to investigate the complex interactions between the individual components and structures involved, such as RPE and photoreceptors. Particularly the normal function of the RPE seems to be fundamental for normal vision and prevention of photoreceptor and resulting vision loss.

Most studies on this matter rely on rodent animal models, which have smaller eyes than humans and the disadvantage of lacking an anatomical macula [11]. Apart from non-primate models, the pig eye offers the highest anatomic similarity to the human eye, with an area of increased cone density within the so-called visual streak [12,13,14]. Neuroretinal explants from pig eyes can easily be obtained via a gentle, pretested method to preserve the structural integrity of the retina [15]. The RPE cells fulfill a variety of essential functions, such as the transport of metabolic end products, ions, and water, enabling an exchange between blood and retina [16,17,18,19]. Furthermore, they provide important nutrients to the photoreceptors and ensure a regular substitution of the retina [19,20]. Based on these multiple tasks in maintaining retinal homeostasis, a co-cultivation with primary RPE cells might enable a better insight into the interaction of those cells with photoreceptor cells. In addition, in vivo animal research is increasingly accompanied by ethical concerns and goes along with high cost and complex legal requirements [21]. Thus, an ex vivo model, which mimics the in vivo situation of RPE cell and neuroretina interaction, seems worthwhile.

The aim of this study was to investigate how primary RPE cells and especially their secreted factors affect ex vivo neuroretina cultures. The co-cultivation of primary porcine RPE (ppRPE) cells and explants indicated a beneficial effect of RPE cells. This facilitates the investigation of the interaction between RPE cells with the neuroretina and moreover enables further studies on neuroretinal diseases and potential therapeutic interventions.

## 2. Materials and Methods

### 2.1. Dissection and Isolation of Primary Porcine RPE Cells

Primary RPE cells were isolated from freshly enucleated pig eyes after obtaining them from the local abattoir and transporting them on ice in an antiseptic solution (Videne antiseptic solution: PBS 1:5). Eyes were trimmed of excess tissue and incubated in 200 U/mL penicillin and 0.2 mg/mL streptomycin solution at 4 °C for 30 min. Eyes were dissected and the neuroretina was carefully removed from the subjacent RPE. Afterwards, each eyecup was incubated in 1 mL 1x Trypsin-EDTA (Merck, Darmstadt, Germany) at 37 °C for 5 min. Next, a papain solution (DMEM/F-12 supplemented with 40 µL DNAse (Worthington, Columbus, OH, USA), 40 µL L-cysteine (Sigma-Aldrich, St. Louis, MO, USA), and 30 U/mL papain (Worthington, Columbus, OH, USA)) was applied for 1 h. Subsequently, the cells were collected by gentle trituration. The ppRPE cells were centrifuged at 3000 rpm for 10 min and then transferred into T25 flasks (Sarstedt, Nümbrecht, Germany) in 5 mL RPE culture medium (DMEM/F12, Gibco Life Technologies, Carlsbad, CA, USA) supplemented with 10% FBS superior (Sigma-Aldrich, St. Louis, MO, USA), 2% penicillin/streptomycin (Sigma-Aldrich, St. Louis, MO, USA) and 1% gentamicin (Sigma-Aldrich, St. Louis, MO, USA). The medium was replaced daily.

### 2.2. Preparation and Cultivation of Neuroretina Explants

Fresh porcine eyes were obtained and dissected as described in Section 2.1. The preparation of the neuroretina explants was performed using the tweezers method as described previously [15]. In brief, after the dissection of the eye, under aseptic conditions, two neuroretina explants were punched out from the visual streak using a dermal punch (Ø = 6 mm, Pmf medical AG, Cologne, Germany). The explant was lifted with tweezers by grabbing the sclera and rotated manually 180 degrees. Then, the explant was placed onto a cell culture insert (Millipore, Burlington, VT, USA).

Retinal explants in one group were co-cultivated with ppRPE cells in 1 mL 1:1 neuroretina medium and RPE culture medium at 37 °C and 5% CO_2_ for eight days. Neuroretina medium: Neurobasal-A medium (Life Technologies, Carlsbad, CA, USA) supplemented with 0.8 mM L-glutamine (Life Technologies, Carlsbad, CA, USA), 2% B27 (Life Technologies, Carlsbad, CA, USA), 1% N2 (Life Technologies, Carlsbad, CA, USA), and 2% penicillin/streptomycin (Sigma-Aldrich, St. Louis, MO, USA). On days zero, one, two, and three, the medium was completely replaced. On days five and seven, half of the medium was exchanged.

### 2.3. Co-Cultivation of ppRPE Cells and Neuroretina Explants

The ppRPE cells in each flask were dissociated with 1 mL Trypsin-EDTA at 37 °C for 5 min. Cells of passage 2 and/or 3 were counted and 50,000 ppRPE cells were seeded per poly-L-ornithine (Sigma-Aldrich, St. Louis, MO, USA) and coated well (12-well plate; Sarstedt, Nümbrecht, Germany). The cells were cultured in an FCS-free RPE culture medium at 37 °C and 5% CO_2_ for seven days. The medium was replaced every second day. The first group consisting of single cultured neuroretina explants served as a control group. For the second group, a freshly prepared neuroretina explant was added, after eight days to each well, where feeder cells and explant were co-cultivated for eight following days at 37 °C and 5% CO_2_ with 1:1 FCS-free RPE culture medium and neuroretina medium. For the third group, explants were cultured in 1:1 FCS-free RPE culture medium and conditioned medium. For this purpose, FCS-free RPE culture medium was collected during the cultivation of ppRPE cells before they became confluent. The conditioned medium was collected twice per week during full medium exchanges of the cultivated ppRPE cells over a period of two weeks. Afterwards, the medium was centrifuged at 3000 rpm for 5 min to remove cell debris. The conditioned medium was collected in sterilized foil-wrapped glass containers and stored at 4 °C until further use. This conditioned medium was used to cultivate the neuroretina samples in the mentioned third group.

**Figure 1:**
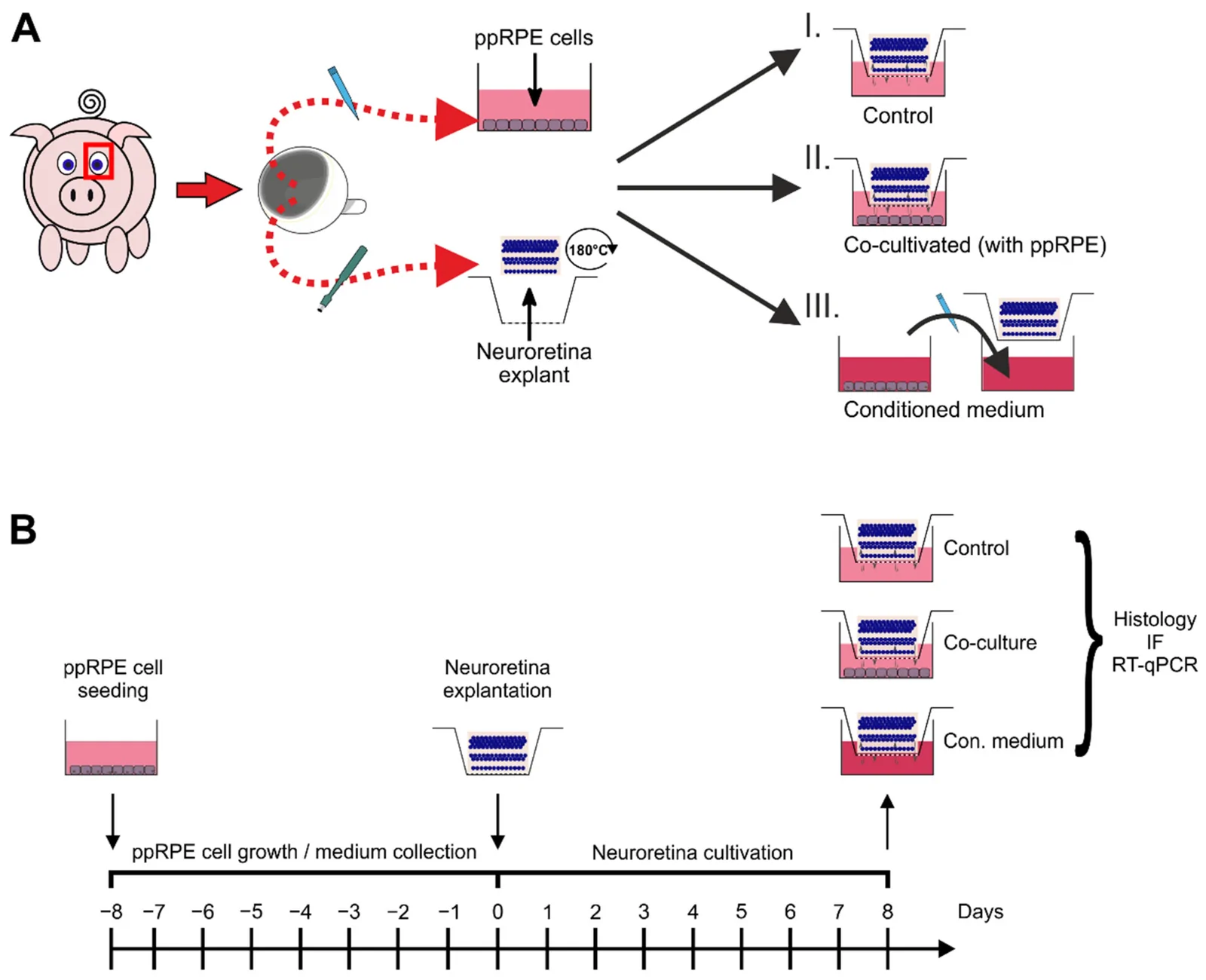
Graphical outline of technique and study design. (**A**) Schematic overview of the three assessed groups named control, co-culture, and conditioned medium. Fresh porcine eyes were used to isolate primary porcine RPE cells (ppRPE). Neuroretina explants were obtained from the visual streak. Single cultured neuroretina explants cultured for eight days served as control (I.). In addition, a co-cultivation with ppRPE cells and neuroretina was performed (II.) and a neuroretina group was cultivated with conditioned ppRPE medium (III.). (**B**) ppRPE cells were seeded on wells eight days before neuroretina explants were prepared. On day zero, neuroretina explants were added to a ppRPE cell feeder layer or cultivated with conditioned ppRPE medium. On day eight, samples from all three groups were collected for histology, immunofluorescence (IF), and RT-qPCR analysis.

In total, three groups were assessed: neuroretina samples alone (control), neuroretina co-cultivated with ppRPE cells (co-culture), and neuroretina explants cultivated in ppRPE-conditioned medium (con. medium; Figure 1A).

### 2.4. Tissue Processing and Preparation for Histology and RT-qPCR

After eight days of cultivation, the neuroretina samples (n = 8/group) were fixed with 4% paraformaldehyde (PFA; Merck) for 15 min. Next, they were drained with 15% sucrose solution (Sigma-Aldrich, St. Louis, MO, USA) for 15 min and 30% sucrose solution for 30 min. In the final step, samples were embedded in NEG-50 Tissue Tek medium (Thermo Fisher Scientific, Waltham, MA, USA) and stored at −80 °C. Then, samples were cut into 10 µm cross-sections via a microtome (Thermo Fisher Scientific, Waltham, MA, USA) and placed on Histobond slides (Paul Marienfeld GmbH & Co. KG, Lauda-Königshofen, Germany) and air-dried at room temperature overnight. On the following day, all slides were fixed in ice-cold acetone for 10 min and stored at −80°C until further processed. Neuroretina samples for RT-qPCR analysis (n = 6/group) were immediately frozen at −80 °C (Figure 1B). Eight samples per group were stained for histology, whereas six samples of each investigated group were analyzed with RT-qPCR.

### 2.5. (Immuno-) Histology

For histological analysis, representative sections of each retina were stained with hematoxylin and eosin (H&E). Following staining, the thickness of the neuroretina [GCL-OS] and the bacillary layer, representing the outer segment layer [OS-ONL], were measured via ZEN 2012-imaging software (blue edition, ZEISS, Oberkochen, Germany). The applied established manual was described before [15].

TUNEL-assay was performed to visualize apoptotic cells. Labeling of apoptotic cells was carried out according to the manufacturer’s instructions (TMR red, Roche^®^, Basel, Switzerland).

To identify different cell types of the retina, specific primary antibodies (Table 1) were used for immunofluorescence staining (IF) [22,23]. First, neuroretina sections were defrosted and dried at 37 °C for 15 min. Next, they were rinsed in PBS and blocked with antisera (goat or donkey) diluted in 0.1–0.2% Triton X-100, PBS (PBST) and 1% bovine serum albumin (BSA). Thereafter, the sections were incubated in a primary antibody solution diluted in PBST at room temperature overnight. This was followed by washing steps with PBS. On the next day, the slides were incubated with corresponding secondary antibodies labeled with Alexa Fluor 488 or Alexa Fluor 555 at room temperature for 1 h. Afterwards, nuclei were specifically counterstained with 0.01 µg/mL 4′,6-diamidino-2-phenylindole (DAPI; Serva Electrophoresis, Heidelberg, Germany). As a no antibody control, the primary antibody was omitted. Lastly, all slides were covered in Shandon mount media (Thermo Fisher Scientific, Waltham, MA, USA).

### 2.6. Microscopy and Image Assessment

Four images per section were taken using an Axio Imager M1 microscope (Zeiss, Oberkochen, Germany). Finally, twelve images per porcine neuroretina explant, of each cultivation condition, were studied. All images were masked and then cut into predefined sections (Corel PaintShop Pro X8; Corel, Ottawa, ON, Canada). C3, DAPI, MAC, Iba1, iNOS, and opsin-labeled cell bodies were counted using the ImageJ plugin “Cell counter” (V1.3u, NIH; Bethesda, MD, USA). For area analysis, an established protocol based on an ImageJ macro was used to quantify PSD-95, rhodopsin, and vGluT1 signal area [24]. Briefly, images were transformed into 32-bit grayscale. To minimize interferences with background labeling, a rolling ball radius was subtracted (Supplementary Table S1). Then, for each picture, a suitable lower and upper threshold was determined and the mean value of the thresholds was used for area analysis (Supplementary Table S1).

In addition, to identify if the order of cell layers in the ONL was still intact, the number of cell layers within the ONL were determined by counting the DAPI-stained cell nuclei. To this end, three white lines across the ONL (left, middle, and right) were drawn. Then, each DAPI^+^ cell which was marked by the vertical lines and was counted. Hence, the number of cell layers was evaluated. Only DAPI+ cells in direct contact with the white bar were counted and considered to represent one cell row.

### 2.7. Quantitative Real-Time PCR (RT-qPCR)

RNA isolation and cDNA synthesis of porcine retina explants were performed, according to the manufacturer’s instructions, with MultiMACS cDNA Kit (Miltenyi Biotec GMBH, Bergisch Gladbach, Germany). For specific primer design, Primer3 software, based on the published GenBank sequence (GenBank: KM035791.1, http://www.bioinformatics.nl/cgi-bin/primer3plus/primer3plus.cgi/ (access date: January 2018–December 2020), was used (Table 2). With the CfX 96 System (Bio-Rad Laboratories Inc., Hercules, CA, USA), RT-qPCR was performed using the SYBR Green PCR kit (Bio-Rad Laboratories Inc., Hercules, CA, USA). A total of 1 ng/µL of cDNA was applied in a reaction volume of 20 µL and the final primer concentration was 100 nM. Each sample was analyzed in a technical replicate. The expression levels of the target genes were normalized against two housekeeping genes *ACTIN β (ACTB)* and *RIBOSOMAL PROTEIN L4 (RPL4)*.

### 2.8. Statistical Analysis

Shapiro–Wilk test was used to test for the normal distribution of data. RT-qPCR data were analyzed using ANOVA, followed by Dunn’s test (GraphPad Prism 8, San Diego, CA, USA). Immunohistological data were analyzed using one-way ANOVA, followed by Tukey’s post hoc test to evaluate differences between the three groups when normally distributed. For non-normally distributed data, the Kruskal–Wallis test followed by multiple comparison was performed for mean ranks of all groups including Bonferroni correction (Statistica; V 13.3; Dell, Round Rock, TX, USA).

A *p*-value of <0.05 was considered statistically significant. The level of significance was set as * *p* < 0.05, ** *p* < 0.01, and *** *p* < 0.001.

## 3. Results

### 3.1. The Presence of ppRPE Cells Preserved the Neuroretina Most Efficiently in Culture

**Figure 2:**
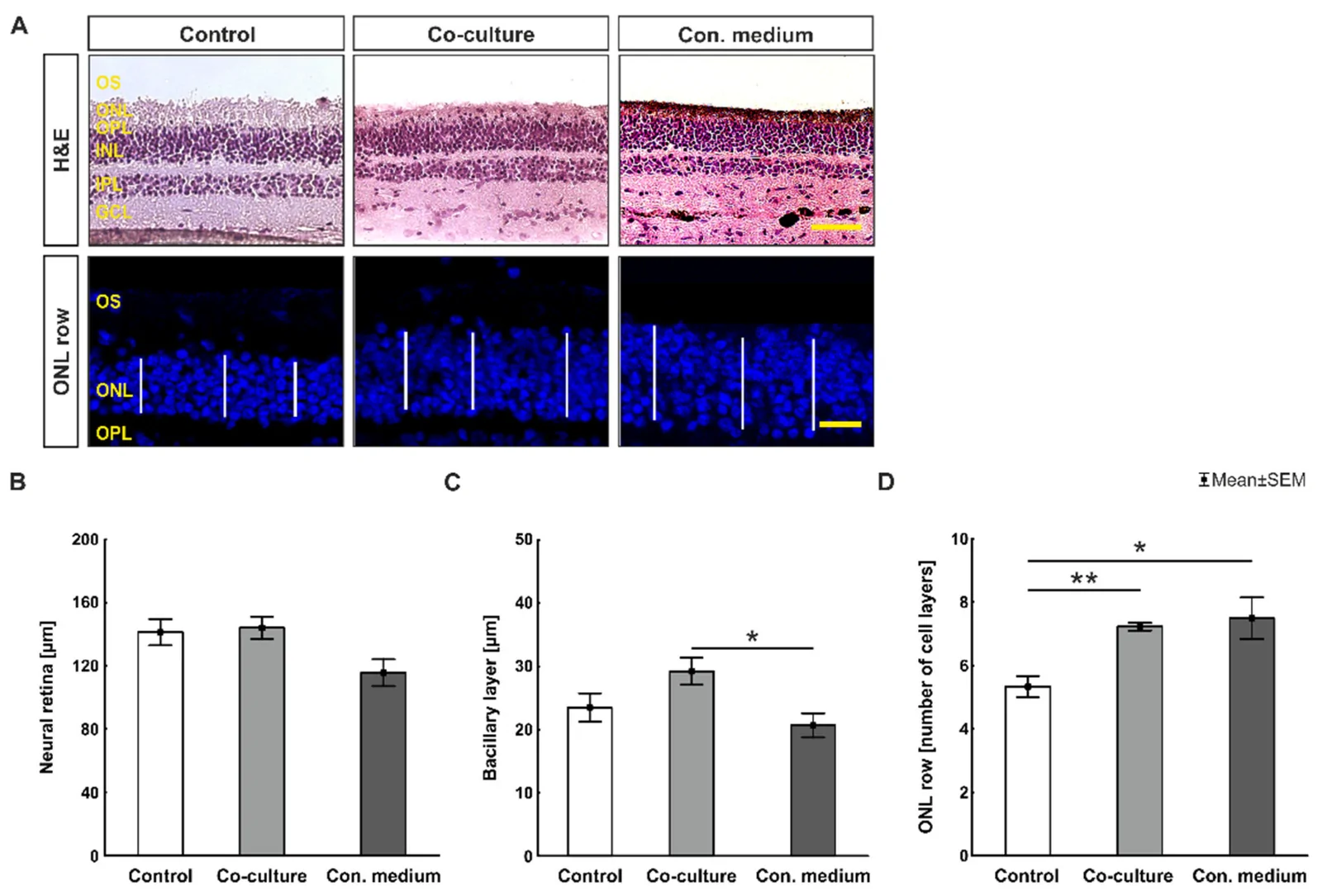
Co-cultivation of the neuroretina with ppRPE cells increased the number of cells in the ONL. (**A**) Upper panel: representative pictures of H&E-stained retina samples of the three groups investigated after 8 days in culture. Bottom panel: representative images of the ONL with DAPI-labeled cell nuclei (blue). To count the cell layers of the ONL, they were marked with three white bars and counted. (**B**) Neuroretina thickness was not altered in the three groups. (**C**) The bacillary layer thickness of the co-culture retinae was comparable to controls, while a significantly thinner bacillary layer was detected in the conditioned medium (*p* = 0.024) groups in comparison to co-culture samples. (**D**) The number of cell layers in the ONL was significantly higher in the co-culture (*p* = 0.003) and conditioned medium group (*p* = 0.030) compared to control retinae. OS, photoreceptor outer segments; ONL, outer nuclear layer; OPL, outer plexiform layer; INL, inner nuclear layer; IPL, inner plexiform layer; GCL, ganglion cell layer. Scale bars: H&E: 50 µm, IF: 20 µm, values are presented as mean ± SEM, n = 8/group, * *p* < 0.05, ** *p* < 0.01.

In order to investigate the structural integrity of the neuroretina, H&E staining of the retinae was performed. The retinal structure and cell layers were preserved without significant distortion in all groups after 8 days in culture (Figure 2A). To analyze the integrity of the neuroretina, the thickness from the retinal ganglion cell layer (GCL) to the outer photoreceptor segments (OS) was measured. Retina thickness of samples from the co-culture group were comparable to control samples (*p* = 0.966). Contrarily, the thickness of conditioned medium retina samples indicated a trend towards thinning compared to co-culture samples (*p* = 0.050, Figure 2B).

In addition, a measurement of the bacillary layer thickness, from outer to inner segment, was performed. The thickest bacillary layer was found in the co-culture group but still comparable to control. In contrast, the bacillary layer of the conditioned medium group retinae was significantly thinner than co-culture ones (*p* = 0.024, Figure 2C).

DAPI^+^ cell rows were counted in the ONL. Compared to the controls, the number of labeled ONL cell nuclei was significantly higher in co-culture (*p* = 0.003) and conditioned medium samples (*p* = 0.030, Figure 2D).

In conclusion, neuroretina and bacillary layer thickness were best preserved in the co-culture group.

### 3.2. Presence of ppRPE Cells Maintains Rods

**Figure 3:**
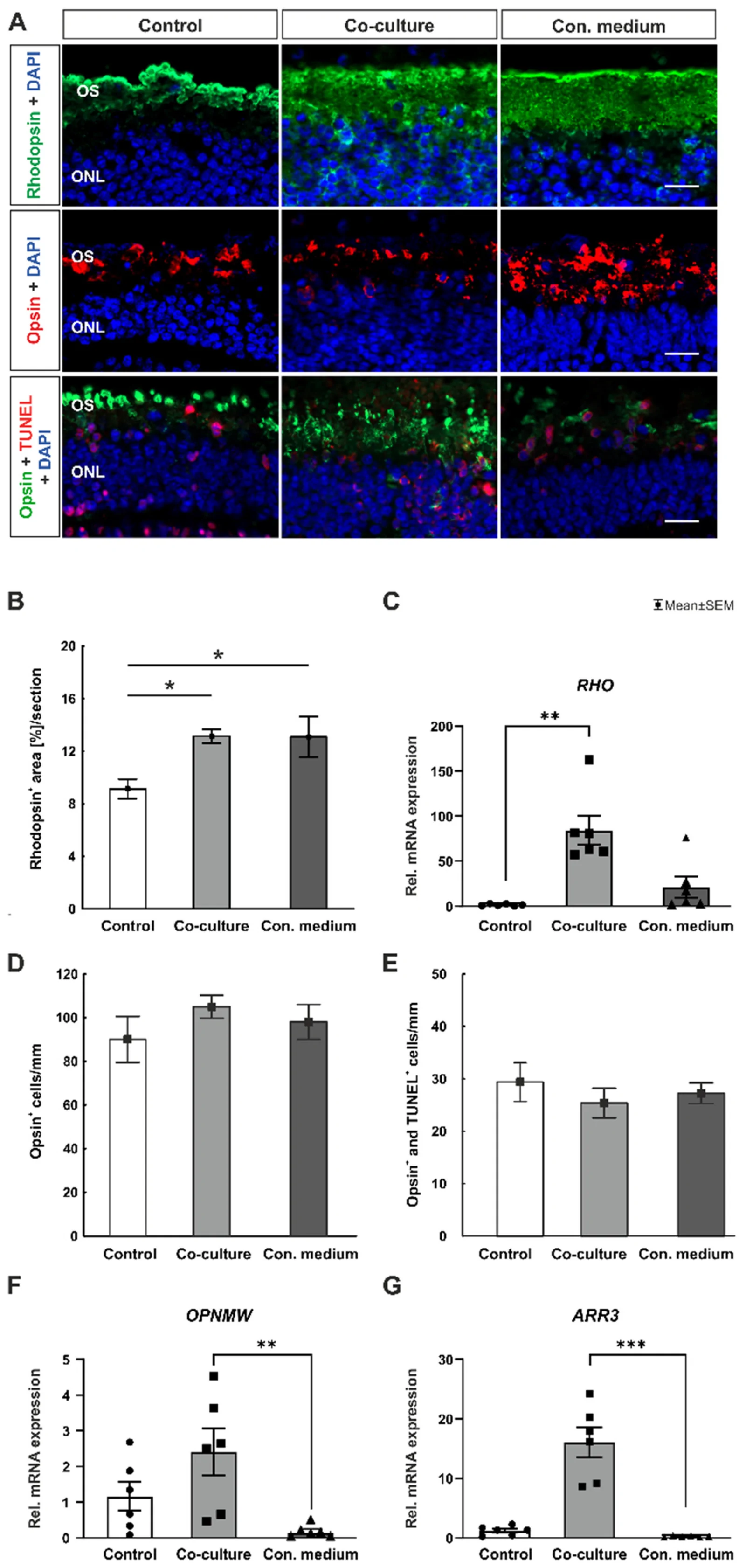
Rods and cones are preserved by co-cultivation with ppRPE. (**A**) Rods were stained with rhodopsin (green), L-cones with opsin (red), and cell nuclei were labeled with DAPI (blue). Additionally, double staining of TUNEL (red) and anti-M/L opsin (green) was performed. (**B**) The rhodopsin^+^ signal area was significantly increased in the co-culture (*p* = 0.033) and conditioned medium group (*p* = 0.035) compared to controls. (**C**) Relative mRNA expression of *RHO* was significantly increased in the co-culture group (*p* = 0.001) compared to control. (**D**) The amount of opsin^+^ cells was comparable in all three groups. (**E**) The number of Opsin^+^ and TUNEL^+^ was even in all three groups. (**F**) The co-culture group had a significantly higher expression of *OPNMW (p* = 0.009) compared to the conditioned medium group. (**G**) *ARR3* expression was increased in co-culture samples in contrast to conditioned medium samples (*p* < 0.001). OS, photoreceptor outer segments; ONL, outer nuclear layer. Scale bar: 20 µm, values are presented as mean ± SEM, (**A**,**B**,**D**,**E**) n = 8/group, (**C**,**F**,**G**) n = 6/group, * *p* < 0.05, ** *p* < 0.01, *** *p* < 0.001.

To assess the preservation of rods and L-cones, retina samples of all three groups were immunohistochemically stained and examined. Rhodopsin staining revealed no differences within the outer and inner photoreceptor segments of marked rod cells (Figure 3A). A significant larger rhodopsin^+^ area was measured in the co-culture samples compared to control (*p* = 0.033) and conditioned medium samples (*p* = 0.035). The rhodopsin^+^ area in conditioned medium retinae did not differ from RPE co-culture (*p* = 1.000, Figure 3B). Additionally, gene expression analysis of RHODOPSIN (RHO) was performed. In co-culture, RHO expression was significantly increased compared to control (*p* = 0.001, Figure 3C).

L-cones were labeled with an opsin antibody. In retina samples of all three conditions, cone cells appeared organized in rows located within the outer photoreceptor segment (Figure 3A). The number of L-cones was comparable between all three groups (*p* > 0.05). Nevertheless, the highest number of opsin^+^ cells was detected in the co-culture group, while the lowest number was found in the control group (Figure 3D).

To detect a specific effect on cone-cell survival, a double staining of TUNEL and anti- M/L opsin was performed. Interestingly the number of opsin^+^ and TUNEL^+^ was equal in all three groups (*p* > 0.05, Figure 3E).

Furthermore, *MEDIUM-WAVE-SENSITIVE OPSIN (OPNMW)* expression was enhanced in the co-culture group in contrast to conditioned medium ones (*p* = 0.009, Figure 3E). Moreover, *RETINAL CONE ARRESTIN-3 (ARR3)* expression was analyzed via RT-qPCR. *ARR3* accumulates in a light-adapted retina, particularly in the outer segments, whereas it is predominantly located within the inner segment of the cones in the dark [25,26,27,28]. A significantly enhanced *ARR3* expression in the co-culture group was detected in contrast to the conditioned medium group (*p* < 0.001, Figure 3F). Although the gene expression of *ARR3* in the co-culture group was markedly increased compared to controls, no statistically significant differences were evident. Due to the variations in the co-culture group, no significant difference in comparison to the control group was detected.

In conclusion, the presence of ppRPE cells resulted in advantageous rod cultivation. While co-culture implies an increased *OPNMW* mRNA expression compared to conditioned medium retinae, the number of opsin^+^ cells was exaggerated in conditioned medium samples.

### 3.3. Co-Cultivation with ppRPE or Conditioned Medium Led to Unaltered Synaptic Density

**Figure 4:**
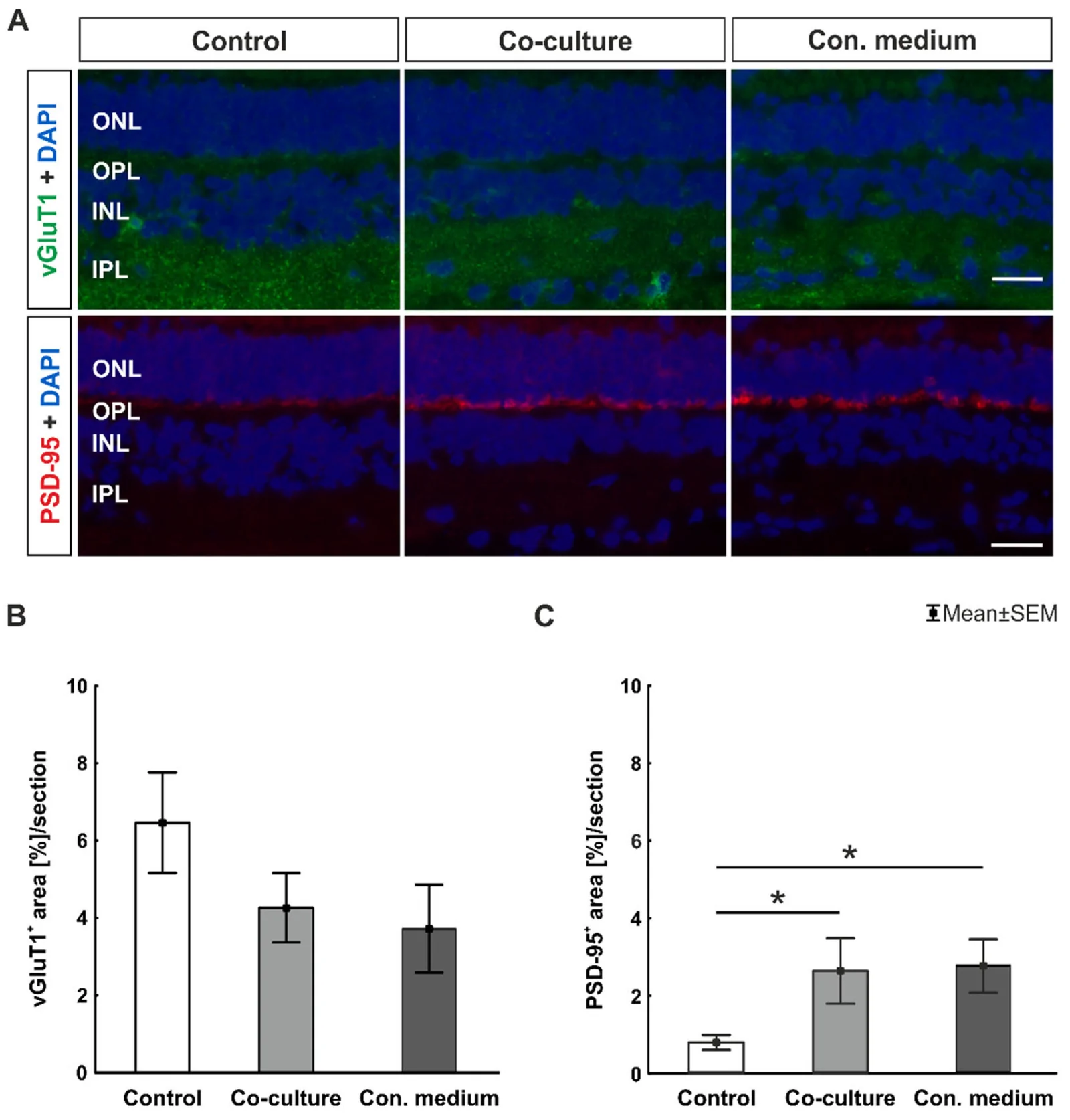
Unaltered synaptic density in cultivated retina. (**A**) To label the uptake of glutamate into synaptic vesicles at presynaptic nerve terminals, vGluT1 antibody was used (green). Whereas the PSD-95 antibody (red) stained the postsynaptic terminals. Cell nuclei were labeled with DAPI (blue). (**B**) vGluT1^+^ signal area was not altered in co-culture or conditioned medium groups compared to controls. (**C**) More PSD-95^+^ signal area was observed in the co-culture (*p* = 0.049) and conditioned medium group (*p* = 0.022) compared to controls. ONL, outer nuclear layer; OPL, outer plexiform layer; INL, inner nuclear layer; IPL, inner plexiform layer. Scale bar: 20 µm, values are presented as mean ± SEM, n = 8/group, * *p* < 0.05.

vGluT1 and PSD-95 markers were used to examine the density and location of synaptic vesicles of neuroretina probes after 8 days in culture (Figure 4A). The glutamate uptake into synaptic vesicles at the presynaptic nerve terminals was detected via vGluT1, while the PSD-95 antibody was used to label postsynaptic density [29,30]. The vGluT1^+^ signal area was comparable to the control in the co-culture (*p* = 0.572) and conditioned medium (*p* = 0.183) group (Figure 4B). Notably, the PSD-95^+^ area was significantly larger in co-culture (*p* = 0.049) and conditioned medium samples (*p* = 0.022) compared to control retinae (Figure 4C).

In summary, no alteration was noted in presynaptic density in the co-culture or conditioned medium groups in comparison to control samples. However, postsynaptic density was significantly increased in the co-culture and conditioned medium compared to control retinae.

### 3.4. No Microglia Activation

**Figure 5:**
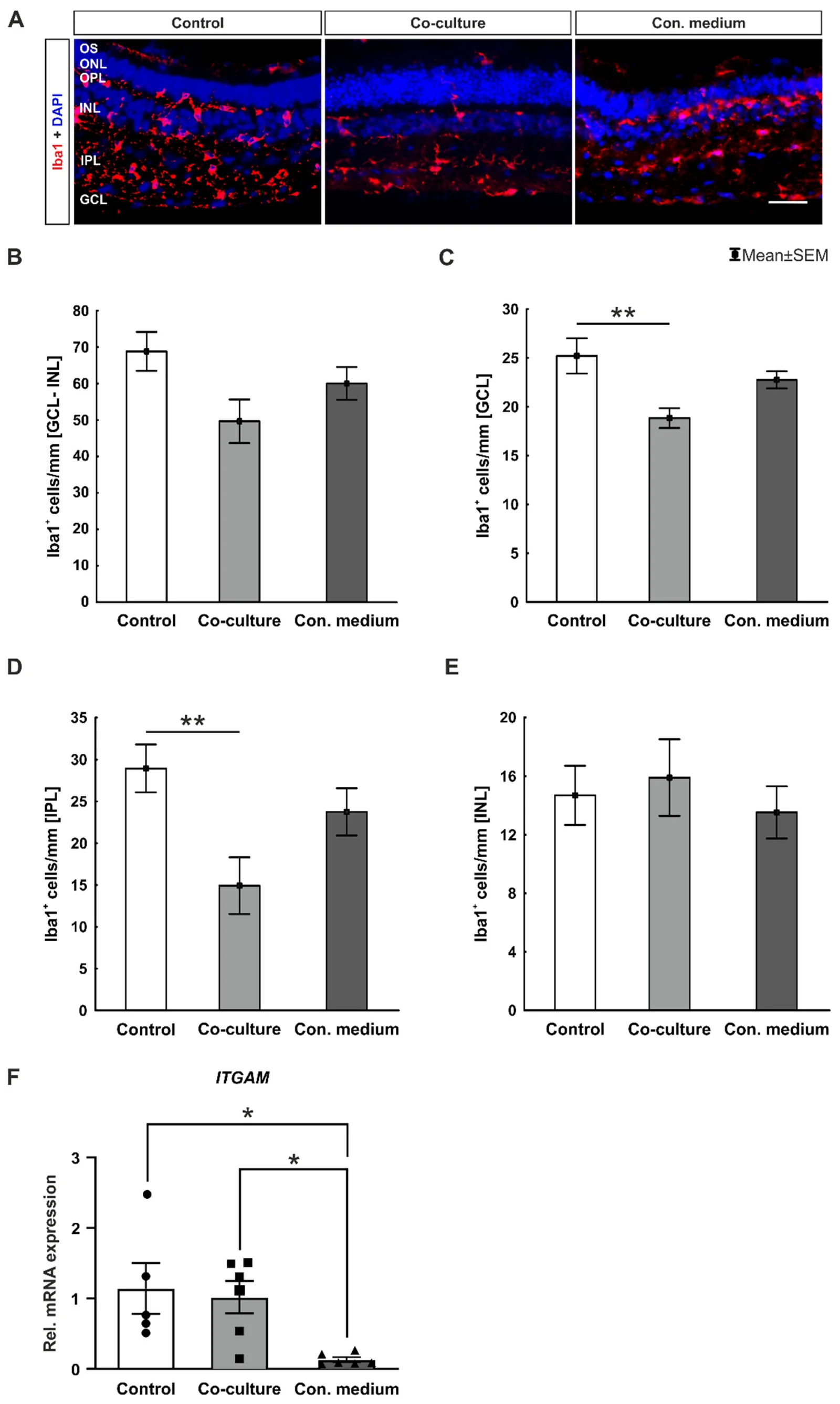
Co-cultivation with ppRPE led to reduced microglial activity in the neuroretina. (**A**) Iba1 antibody was used to label microglia (red). Cell nuclei were labeled with DAPI (blue). (**B**) The number of Iba1^+^ cells in the GCL to INL were similar in all three groups. (**C**) The number of Iba1^+^ cells in the GCL was reduced in the co-culture group compared to controls (*p* = 0.005). (**D**) Analysis of the IPL revealed significantly lower Iba1^+^ cell counts in co-culture group than in controls (*p* = 0.008). (**E**) The number of Iba1^+^ cells in the INL was comparable in all groups. (**F**) Relative mRNA expression of *ITGAM* was significantly decreased in the conditioned medium group compared co-culture (*p* = 0.022) and control samples (*p* = 0.023). OS, photoreceptor outer segments; ONL, outer nuclear layer; OPL, outer plexiform layer; INL, inner nuclear layer; IPL, inner plexiform layer; GCL, ganglion cell layer. Scale bar: 20 µm, values are presented as mean ± SEM, (**A**–**E**) n = 8/group, (**F**) n = 6/group, * *p* < 0.05, ** *p* < 0.01.

The microglial activity was determined via Iba1 staining and *integrin alpha M* (*ITGAM)* expression. Iba1^+^ cells were counted in all retina samples after 8 days of cultivation in the neuroretina and distinct layers such as GCL, IPL, and INL (Figure 5A–D). Similar cell counts were noted in the conditioned medium and control group (*p* = 0.512). Interestingly, the number of Iba1^+^ cells, within the area from GCL to INL, were comparable in all three groups (Figure 5B).

To further investigate the microglia activity, a detailed counting of positive-labeled cells in the individual retina layers was performed. Concerning the GCL, a significantly decreased number of Iba1^+^ cells was observed in co-culture samples compared to the control group (*p* = 0.005; Figure 5C). Regarding the IPL, a significantly reduced number of Iba1^+^ cells in co-culture retinae was noted in contrast to control ones (*p* = 0.008; Figure 5D). However, no difference in Iba1^+^ cell numbers was observed within the inner nuclear layer (INL) in all three groups (Figure 5E). Furthermore, iNOS^+^ cells together with Iba1^+^ cells were used to visualize active microglia (Supplementary Figure S1). In general, no effects of co-culture or conditioned medium could be observed in regard to numbers of active microglia in comparison to control (Supplementary Figure S1B–E). There was no alteration in the amount of iNOS^+^ cells and iNOS^+^ + Iba1^+^ cells in the retina from the GCL to INL and distinct counted layers (GCL, IPL, INL) between all examined groups (Supplementary Figure S1F–I).

The integrin alpha M was expressed in macrophages and microglia [31]. *ITGAM* mRNA expression in the conditioned medium group was significantly reduced compared to the control (*p* = 0.023) and co-culture group (*p* = 0.022, Figure 5F). Moreover, similar levels for *NITRIC OXIDE SYNTHASE 2* (*NOS2*) mRNA expression were detected in all three groups (Supplementary Figure S1J).

In conclusion, a significant reduction in Iba1^+^ cell numbers was detected in the neuroretina and in the detailed analysis of GCL and IPL in the co-culture retinae compared to the control ones.

### 3.5. No Complement System Activation In Vitro

**Figure 6:**
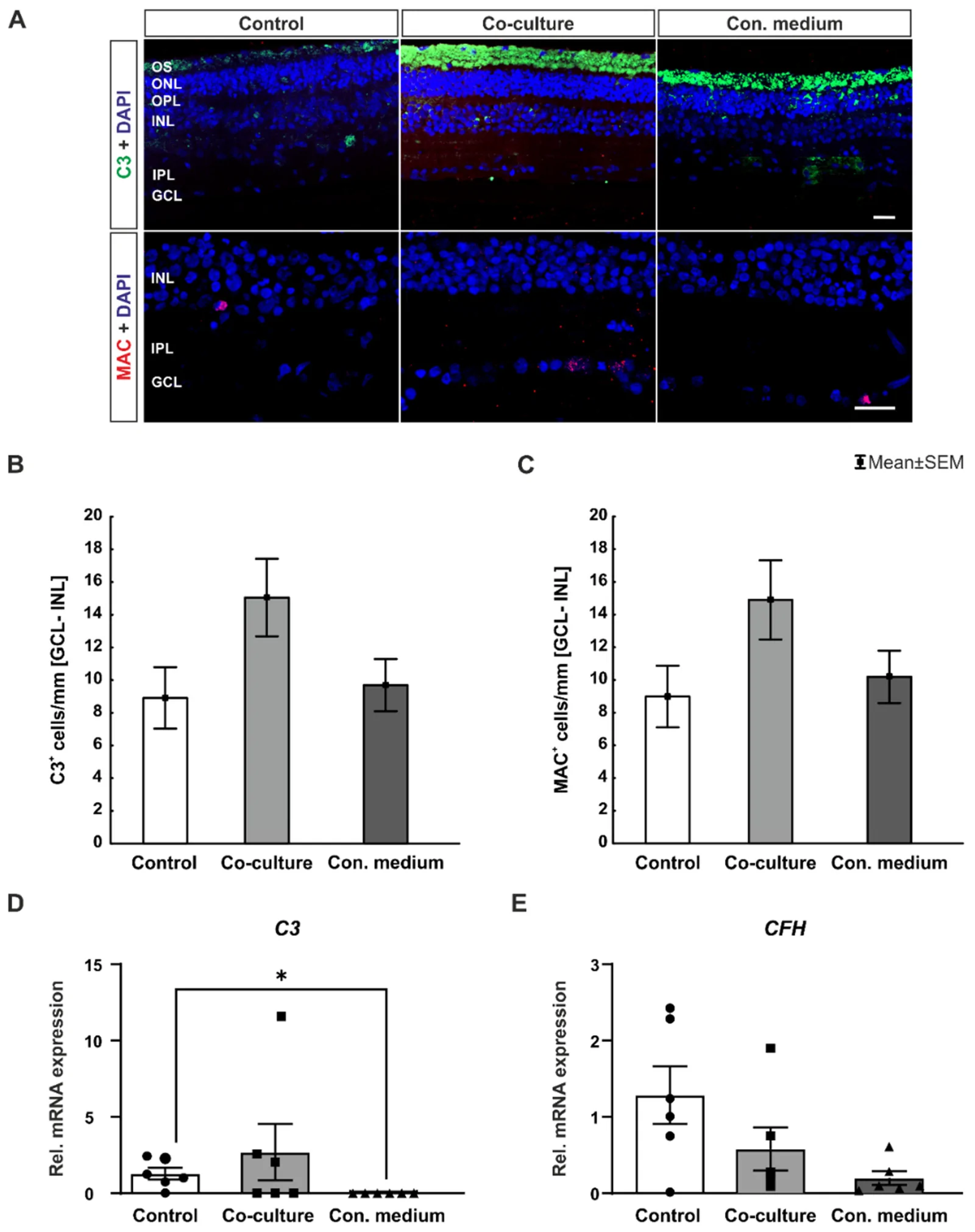
Unaltered complement activity. (**A**) To evaluate the complement factors, complement component 3 (C3, green) and the membrane attack complex (MAC, red) were labelled. Cell nuclei were marked with DAPI (blue). (**B**) The number of C3^+^ cells was comparable in the GCL–INL in all groups. (**C**) No differences were noted regarding MAC^+^ cell numbers in GCL–INL in all groups. (**D**) Relative mRNA expression of *C3* was significantly decreased in the conditioned medium group compared to control (*p* = 0.021). (**E**) Relative mRNA expression of *CFH* showed no significant differences between groups. OS, photoreceptor outer segments; ONL, outer nuclear layer; OPL, outer plexiform layer; INL, inner nuclear layer; IPL, inner plexiform layer; GCL, ganglion cell layer. Scale bars: 20 µm, values are presented as mean ± SEM, (**A**–**C**) n = 8/group, (**D**–**E**) n = 6/group, * *p* < 0.05.

The complement system can be triggered through three pathways. All three unite in the activation of complement component 3 (C3), which was analyzed in this study together with the membrane attack complex (MAC)—the terminal component of the complement system (Figure 6A). Concerning C3 cell counts, no changes were identified in all retina samples after 8 days (Figure 6B).

The number of MAC^+^ signals was equivalent in all three groups (Figure 6C).

In addition, *C3* was also examined via RT-qPCR. The *C3* mRNA expression was significantly reduced in the conditioned medium group compared to control (*p* = 0.021, Figure 6 D), while the *COMPLEMENT FACTOR H* (*CFH)* expression was slightly, but not significantly, reduced compared to controls (Figure 6E).

In summary, the number of C3^+^ and MAC^+^ cells, as well as the CFH expression, was unaltered in the co-culture and conditioned medium group in comparison to the control group; meanwhile, the *C3* expression was significantly diminished in the conditioned medium group.

### 3.6. Pro-Inflammatory Cytokines were Upregulated in Co-Culture and Conditioned Medium Samples

**Figure 7:**
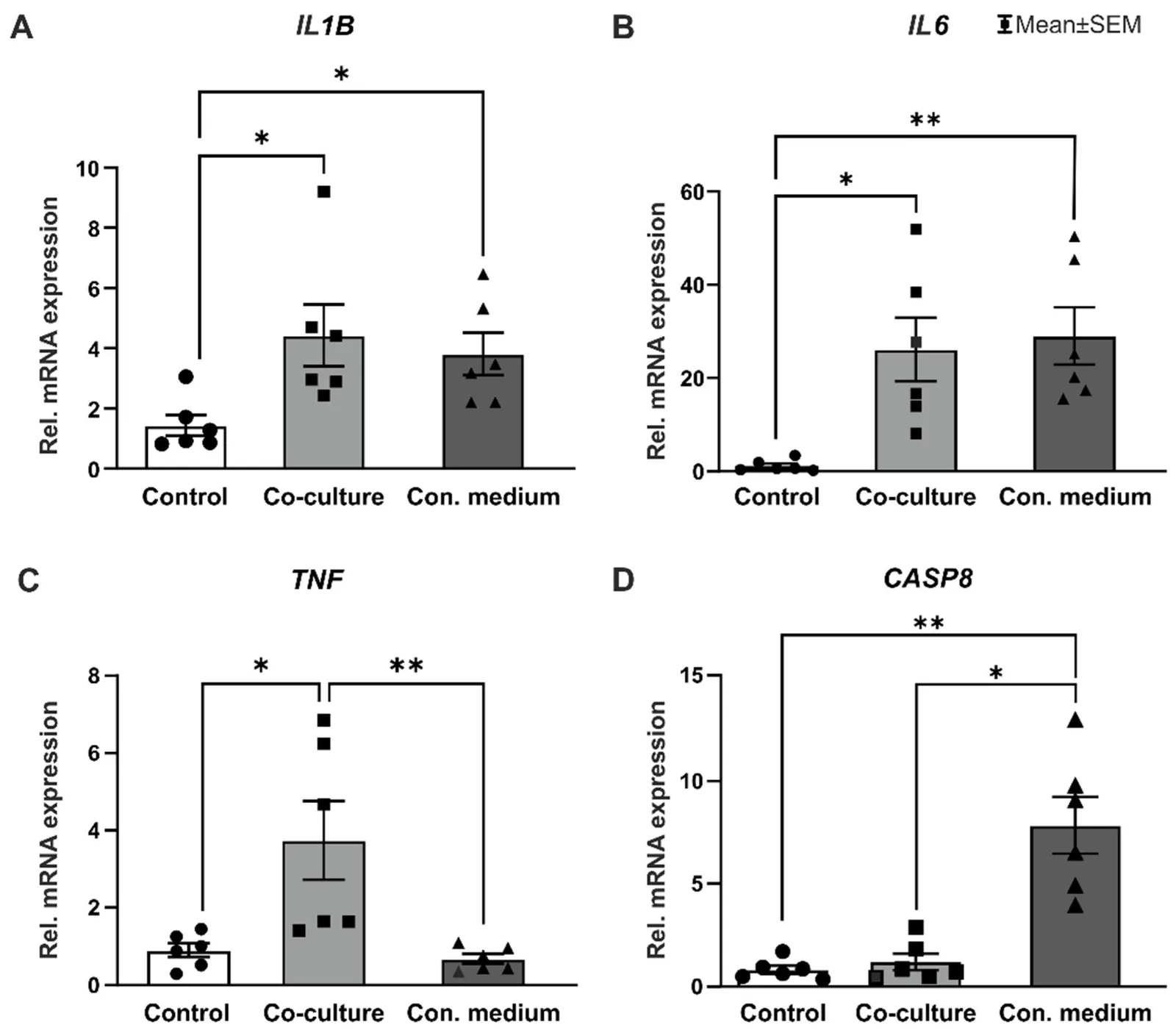
Co-cultivation with ppRPE leads to the upregulation of pro-inflammatory cytokines. (**A**) Relative mRNA expression of *IL1B* was significantly enhanced in co-culture (*p* = 0.028) and conditioned medium samples (*p* = 0.045) compared to the control group. (**B**) *IL6* mRNA expression was significantly increased in co-culture (*p* = 0.018) and medium (*p* = 0.006) groups compared to control. (**C**) In co-culture group, relative mRNA expression of *TNF* was significantly upregulated compared to control (*p* = 0.039) and medium (*p* = 0.004) samples. (**D**) *CASP8* mRNA expression was significantly increased in medium group compared to control (*p* = 0.006) and co-culture group (*p* = 0.018). Values are presented as mean ± SEM, n = 6/group, * *p* < 0.05, ** *p* < 0.01.

Inflammatory and immunological processes were analyzed in all three cultivation groups. Pro-inflammatory cytokines, such as interleukin (IL) 1β, IL6, and tumor necrosis factor (TNF), are predominantly produced by activated macrophages in response to inflammation [32]. Notably, a significant upregulation of *IL1B* expression was noted in the co-culture samples compared to the control group (*p* = 0.028). Moreover, *IL1B* expression was significantly increased in the conditioned medium retinae in contrast to controls (*p* = 0.045, Figure 7A). Elevated *IL6* expression was detected in the co-culture retinae compared to the control group (*p* = 0.017). Gene expression levels were increased in the conditioned medium and co-culture samples in contrast to the control (*p* = 0.006, Figure 7B). Moreover, the *TNF* expression was upregulated in the co-culture (*p* = 0.048) compared to the control and conditioned medium samples (*p* = 0.003, Figure 7C). 

Finally, the expression of *CASP8* (*CASPASE 8*), which is an initiator caspase of the extrinsic cell death–signaling pathway, was examined. Strikingly, the *CASP8* expression was significantly increased in the conditioned medium samples compared to the control (*p* = 0.006) and co-culture group (*p* = 0.018, Figure 7D).

In conclusion, pro-inflammatory cytokines *IL1B*, *IL6*, and *TNF* were all significantly upregulated in the co-culture groups in contrast to the control retinae. Additionally, cytokines *IL1B* and *IL6* were significantly upregulated in the conditioned medium samples compared to the control retinae. The expression of *CASP8* was significantly enhanced in the conditioned medium samples compared to the control ones.

## 4. Discussion

The etiology and pathology of multifactorial retinal diseases such as AMD are still poorly understood. AMD is characterized by degeneration involving the retinal cone and rod photoreceptor, RPE, as well as adjacent Bruch’s membrane and modifications in choroid [33,34]. Since human donor eyes are only of limited availability, porcine eyes might be an alternative for organ culture models as they can be obtained as a waste product from the food industry. Previously, rodent eyes were used to establish models. However, porcine eyes provide a much closer homology to human eyes in diverse anatomical and physiological points [11,35,36]. Subsequently, neuroretina explants were obtained from the visual streak of each porcine eye to achieve a high similarity to the human fovea [37,38]. Ghareeb et al., as well as Schnichels et al., reviewed and summarized the possibilities and benefits of advanced co-culture techniques, which emulate the RPE–photoreceptor and RPE–Bruch’s–choriocapillaris interactions. They pointed out that there are potential questions to address, especially in relation to disease modeling and the optimization of regenerative cell therapies for retinal degeneration [21,39]. Our aim was to establish an ex vivo porcine neuroretina—RPE co-cultivation model. Thereby, allowing the investigation of associated pathological changes and the interdependency of neuroretina and RPE cells.

### 4.1. Best Preservation of Neuroretina in ppRPE Co-Cultivation

To characterize our novel model and explore screening possibilities for degeneration and interaction processes, we first examined the structural integrity of the neuroretina explants to ensure that the new cultivation approaches preserve the complex structures and cell–cell interactions of this delicate tissue. Retinal cryosections were stained with H&E. After 8 days in culture, the co-culture and conditioned medium samples displayed a maintained multi-layered retinal structure and similar neuroretina thickness compared to the controls. Interestingly, a slight reduction in neuroretina thickness was observed in the conditioned medium in comparison to the co-culture samples. It is documented that in single neuroretina explants, the thickness of the INL and ONL decreases over time during the cultivation period of eight days [15,40].

Moreover, the bacillary layer of the conditioned medium retinae was significantly thinner in contrast to the co-culture group. Both results taken together indicate better conservation of the neuroretina morphometry through a co-cultivation with a ppRPE feeder layer.

In addition, the number of DAPI^+^ cells in the ONL was comparable in the co-culture and conditioned medium group—both were elevated in comparison to the control group. Based on these findings, better viability and structural integrity became evident in the co-culture samples—being advantageous in comparison to the conditioned medium cultivation. This beneficial effect of RPE cell co-cultivation on the preservation of retinal photoreceptor cells has also been observed by Di Lauro et al. [40]. Subsequently, it can be assumed that ppRPE secreted factors lead to this neuroprotective effect. These factors secreted by the RPE cells in vivo fulfill various important functions [19]. The RPE primarily secretes various growth factors such as ciliary neurotrophic factor (CNTF), fibroblast growth factors (FGF-1, FGF-2, and FGF-5), insulin-like growth factor-I (IGF-I), platelet-derived growth factor (PDGF), pigment epithelium-derived factor (PEDF), and transforming growth factors (TGF-β) and VEGF [19]. Additionally, Kaempf et al. noted a lower level of apoptosis in co-cultured explants in comparison to single neuroretina samples after 3 days. They analyzed a porcine co-culture system consisting of neuroretina explants cultured together with an RPE–choroid layer [41]. Having observed that ppRPE co-cultivation preserved bacillary layer thickness, this layer was examined more closely. In particular, rods and cones were examined as they are located in this layer. The rhodopsin^+^ area was significantly larger in the co-culture and conditioned medium group, whereas the *RHO* expression was solely increased in the co-culture samples. The number of opsin^+^ cells was comparable in all groups, while the *OPNMW* expression was significantly upregulated in the co-culture samples compared to the conditioned medium ones. In addition, *ARR3* expression, a marker gene for cone photoreceptors, was significantly enhanced in the co-culture samples compared to the conditioned medium. Previous studies, using a co-cultivation of porcine neuroretina explants and ARPE-19 feeder layer, noted an improvement on photoreceptor survival and the preservation of ONL integrity [42,43]. This protective effect of co-cultivation was also visible in our study, rods seemed to especially benefit from the ppRPE feeder layer as we detected a greater rhodopsin^+^ area and an increase in *RHODOPSIN* expression in this group. However, a preservation of cone mRNA expression in contrast to the control and conditioned medium group was found. Nevertheless, on a cellular level, no difference was demonstrated in the number of cone cells when comparing all groups. Notably, the preservation of rod and cones seemed to be slightly more prominent in the co-culture group than in the conditioned medium group. Kolomeyer et al. examined trophic factor secretion profiles of fetal and adult RPE cells. In comparison, the fetal RPE-conditioned medium had significant increased concentrations of vascular endothelial growth factor-A (VEGF-A), brain-derived neurotrophic factor (BDNF), and pigment epithelium-derived factor (PEDF) and significantly lower concentrations of leukemia inhibitory factor (LIF), basic fibroblast growth factor (bFGF), and nerve growth factor (NGF) [44]. The fetal RPE-conditioned medium especially showed the potential of advancing porcine retina survival. Via iTRAQ analysis, they identified VEGF-A and PEDF as key RPE-derived factors in preserving retinal viability. Notably they could also show a significantly better porcine retinal survival and preservation when explants were co-cultivated with fetal RPE cells than only conditioned fetal RPE medium [44]. In accordance with their study, we noted a more positive effect in the maintenance of the retinal explant when co-cultured with RPE cells instead of solely conditioned medium. Nevertheless, Kolomeyer et al. also demonstrated a better preservation of the outer nuclear layer and diminished photoreceptor axon retraction by culturing porcine retina explants in RPE-conditioned medium. In comparison to basal medium, the RPE-conditioned medium reduced porcine retinal death by 17–34% (*p* < 0.05). Indeed, it seems to be a concentration-dependent effect. The data of Kolomeyer et al. indicated that PEDF is a key factor of neuroretinal survival [45]. Hence, their results do not show a toxic effect of conditioned medium on retinal preservation. In summary, the presence of the RPE cells has a much better effect on the maintenance of the neuroretinal explants than the conditioned medium or solely basal medium. However, the fetal RPE-conditioned medium especially showed a beneficial effect compared to the basal medium. Therefore, we might need to adjust the concentration of the RPE-conditioned medium and PEDF to see more protective effects on the maintenance of the neuroretina. Nonetheless, the minor effect in our study of the RPE-conditioned medium on retinal explant survival, in contrast to RPE co-cultivation, could be based on the fact that we used adult RPE cells to collect the RPE medium instead of fetal ones.

### 4.2. Altered Postsynaptic Transmission in Co-Culture and Conditioned Medium Conditions

vGluTs are necessary to sequester glutamate into synaptic vesicles for glutamatergic neurotransmission [46]. Photoreceptor and bipolar cells are the principal glutamatergic neurons in retina transferring visual signals to the RGCs. Terminals of photoreceptors and bipolar cells were specifically labeled via anti-vGluT1 [47,48]. In the present study, no difference in the vGluT1^+^ area was noted in all three groups. The PSD-95 protein is expressed in cone and rod photoreceptor synaptic terminals [49,50,51]. Johansson et al. described the PSD-95 staining pattern as a distinct continuous band in the OPL and thin immunolabeled microglial processes close to the mentioned rod and cone terminals in normal porcine retina [52]. Interestingly, this observation corresponds to our images (Figure 4A). We detected a difference in postsynaptic density in the co-cultured and conditioned medium retinae in comparison to the single cultured neuroretina samples. Moreover, Johansson et al. cultured retina samples and noted a patchy PSD-95 staining pattern. However, such a patchy pattern was not visible in our co-culture group. Strikingly, they could find immunolabeled microglia cells located within those PSD-95 staining free gaps [52], indicating that microglia are essential for synaptic functionality and integrity in retina, as it was previously described for the central nervous system [53].

### 4.3. Lower Microglia Numbers in Co-Culture Retinae

A special characteristic of the retina is its immune privilege. This results in specific modifications for injury control and immune regulations [54]. Microglia cells represent the sentinel cells of the innate immune response within the retina [55]. In their ramified state, they surveil their environs to identify potential destructive signals. In response to mentioned danger signals, ramified microglia transform into a highly mobile amoeboid-activated state and exert a pro-inflammatory phenotype. In this context, microglia are essential for the protection and homeostasis of the retina. However, microglial overactivation is suggested as a possible trigger of diverse retinal diseases and degeneration [56]. The inhibition of microglial function caused an accelerated degeneration of photoreceptor cells through debris accumulation and apoptosis [57,58]. Our current study showed a significantly reduced number in the Iba1^+^ microglial cells of the retina from GCL–INL as well as in the GCL and the IPL in co-culture retinae compared to control samples. Johansson et al. noted activated microglia in the cultured porcine retina. In more detail, Iba1 or CD11b immunoreactive microglia showed an alteration in morphology and a localization in proximity to photoreceptor synapses in the OPL [52]. Ozaki et al. identified a microglia activation in a Rho−/− model. The activated microglia infiltrated the outer retina simultaneously with the onset of photoreceptor degeneration [59]. Makabe et al. demonstrated such infiltration into the OS via the ONL in a retinitis pigmentosa model. Moreover, in this study, no effects on the number of active microglia could be detected despite the differing cultivations of the three groups. This supports the assumption that our co-cultivation model provides an option to achieve a preservation of the delicate photoreceptor cells in vitro. Under pathological conditions, iNOS was produced and caused a more severe pathophysiological response aiming in retinal degeneration [60,61,62]. In the current study, we did not detect any alteration in the amount of iNOS^+^ and iNOS^+^ + Iba1^+^ cells in the neuroretina and distinct layers in the co-culture and conditioned medium samples compared to the control ones. Mueller-Buehl et al. demonstrated a better preservation of porcine retina samples in an organotypic oxidative stress model by adding an iNOS-inhibitor [62]. The lacking induction of iNOS and microglial activation might indicate homeostasis of the neuroretina in culture.

Interestingly, the *ITGAM* expression was diminished solely in the conditioned medium group in our study. Generally, an activation of microglia is associated with many retinal diseases, but their exact contribution to the pathogenesis is not yet known [39]. In the present study, we aimed to characterize the structural integrity of the neuroretina explants in culture with the presence of RPE cells or conditioned medium without the addition of any degenerating reagents or stressors. The absence of microglia activation indicates that the used cultivation method does not stimulate a retinal immune response.

### 4.4. No Detectable Complement System Activation

The complement system is composed of numerous proteins. One of those is CFH, as a key regulator, it maintains the ideal C3 level in circulation. A local source of CFH expression within the human eye is the RPE [63,64]. Hoh Kam et al. investigated the role of C3 in retinal health via a C3−/− mice model. They demonstrated that C3−/− knockout mice display unique outer retinal features induced by regional failures of phagocytosis. This results in a local subretinal accumulation of C3 within outer segments. Hence, C3 is an important key player in protecting the aging mouse retina [65]. Interestingly, the *CFH* expression in our data was comparable in all three groups. CFH binds to C3b. In this study, no alteration was detected in the number of C3^+^ cells, even though the *C3* expression was significantly diminished in retina samples cultured with the conditioned medium. In addition, we did not note changes in the number of MAC^+^ cells. Maliha et al. demonstrated an activation of the complement cascade via the classical pathway resulting in significantly higher C3^+^ and MAC^+^ cell numbers in response to induced hypoxia in a porcine organotypic degeneration model [66]. Considering that in our model, no stressors were used, a lacking activation of the complement cascade is advantageous and desired as the activation of the complement system contributes to the production of other inflammatory mediators and can, therefore, encourage retinal injuries and damage.

### 4.5. Pro-Inflammatory Cytokines Are Upregulated in Co-Cultivation

An inflammation is a response to exogenous or endogenous harmful stimuli to protect and ensure the function of a tissue. Nevertheless, this inflammatory response has to be well regulated. As already mentioned, an overactivation could lead to secondary retinal damage and further enhance neuronal and vascular degeneration [67]. One of the many functions of RPE cells is the preservation of the immune privilege by forming the blood–retina barrier [19]. In regard to an inflammatory response, the RPE itself can be active as a sentinel and promote a pro-inflammatory environment [68]. Interestingly, we noted an upregulation of *IL1B*, *IL6*, and *TNF* in retinae co-cultivated with ppRPE cells or in the conditioned medium. Although neuroprotective abilities of co-cultivations became evident, upregulation of these cytokines can be induced by oxidative stress of the retina [69]. For example, patients with diabetic retinopathy show increased levels of IL1β and TNFα in vitreous humor and serum [70]. Additionally, similar regulation profiles for these pro-inflammatory cytokines were observed in RD patients, indicating that those evaluated levels could occur by the separation of neuroretina from RPE [71,72]. On the one hand, IL1β is linked to various eye diseases such as diabetic retinopathy and retinal detachment [73,74]. On the other hand, it is known to promote a neuroprotective influence in mouse retinae, in response to excitotoxic damage [75]. Similar properties are described in the literature for IL6, which is elevated in various mentioned retinal disorders, such as retinal detachment, diabetic retinopathy, and age-related macular degeneration [76,77]. Interestingly, a neuroprotective effect was visible in the retina and in particular photoreceptors [78,79]. The same applies to TNFα, which is also contributing to the development of different neurodegenerative diseases [80]. Furthermore, in the literature, a neurotoxic effect on retina cells was noted for IL1β, IL6, and TNFα due to RPE cell culture, which has been inflammatorily activated [81]. Dietrich et al. observed a secretion of cytokines by the RPE in an inflammatory environment. They noted a regulatory effect of ppRPE cells on microglia cells in culture in order to accomplish RPE’s function to protect the photoreceptor cells [81]. These data are in accordance with our detected upregulated pro-inflammatory cytokines. Taken together, this indicates that the source of the secreted pro-inflammatory cytokines is likely to be the RPE feeder layer, as microglia activation in the retina is nearly absent in our culture model without the ppRPE or conditioned medium.

We also examined the *CASP8* expression, which is a key protease of the extrinsic cell death pathway [82]. Interestingly, *CASP8* expression was solely upregulated in retinae samples cultivated with the conditioned medium. This indicates an initiation of the extrinsic apoptosis pathway in retinae cultivated with the conditioned medium in contrast to the control and co-culture retinae with ppRPE. This result could explain the poorer preservation of the neuroretina in the conditioned medium group in contrast to the co-culture group. The RT-qPCR is a straightforward and quantitative method which enables the detection of various cytokines using small sample amounts. Nevertheless, it needs to be mentioned that the RT-qPCR has a major disadvantage and limitations, as the presence of RNA does not always precisely reflect protein levels. Hence, to analyze the secretion of cytokines, it needs to be noted if the cytokine is regulated at the translational level or post-translationally [83,84,85]. After all, although PCR is a reliable and highly sensitive method to identify interesting candidates, for further analysis, another method will be implemented.

## 5. Conclusions

Our co-cultivation model is designed to answer questions concerning the impact of RPE cells on the neuroretina. In particular, the co-cultivation of ppRPE cells and porcine neuroretina explants showed a beneficial effect on retinal structure and integrity, preserving retinal layer thickness and rod photoreceptor cells.

Furthermore, no microglia and complement system activation was detected by the co-cultivation with the ppRPE cells or conditioned medium. In addition, the co-cultivation with the ppRPE or conditioned medium led to an upregulation of pro-inflammatory markers, whose origin seems to be the RPE cells itself. Although the inflammatory features need to be investigated in more detail, this approach could be a first step towards a co-cultivation system.

## Figures and Tables

**Table 1:** List of used primary and secondary antibodies.

Primary Antibodies				Secondary Antibodies			
Antibody	Catalogue Number/Clone ID	Company	Dilution	Antibody	Catalogue Number	Company	Dilution
Anti-C3	CL7334Ap	Cedarlane	1:500	Goat anti-rabbit Alexa Fluor 488	A11008	Invitrogen	1:500
Anti-C5b-9 (MAC)	HM3033	Biozol	1:100	Donkey anti-mouse Alexa Fluor 555	ab150106	Abcam	1:500
Anti-Iba1	234006	Synaptic System	1:400	Donkey anti-chicken Cy3	AP194C	Milipore	1:500
Anti-iNOS	PA1-036	Thermo Fisher	1:50	Donkey anti-rabbit Alexa Fluor 488	711-547-003	Jackson Immuno Research	1:600
Anti-M/L opsin	AB5405	Millipore	1:1200	Donkey anti-rabbit Alexa Fluor 488	711-547-003	Jackson Immuno Research	1:600
Anti-PSD-95	CP35	Calbiochem	1:300	Donkey anti-mouse Alexa Fluor 555	ab150106	Abcam	1:500
Anti-rhodopsin	ab3267	Abcam	1:400	Goat anti-mouse Alexa Fluor 488	A-11029	Invitrogen	1:500
Anti-vGluT1	135316	Synaptic System	1:100	Donkey anti-chicken Alexa Fluor 488	703-545-155	Jackson Immuno Research	1:500

**Table 2:** List of used RT-qPCR primers.

Gene	Oligonucleotides 5′ 3′	GenBank Accession Number	Amplicon Size
*ACTB for* *ACTB rev*	CTCTTCCAGCCTTCCTTC GGGCAGTGATCTCTTTCT	XM_021086047.1	178
*ARR3 for* *ARR3 rev*	TGACAACTGCGAGAAACAGG CACAGGACACCATCAGGTTG	NM_214345.1	157
*C3 for* *C3 rev*	ACAAATTGACCCAGCGTAGG GCACGTCCTTGCTGTACTGA	NM_214009.1	285
*CASP8 for* *CASP8 rec*	GCCCAGATCTCTGCCTACAG CAGGGCCTTGTTGATTTGTT	XM_021074710.1	227
*CFH for* *CFH rev*	GTGTGTGGTGAAGACGGATG GGGTGGAGCACAGGATTTTA	NM_214281.2	248
*GFAP for* *GFAP rev*	GGAGAAGCCTTTGCTACACG TCTTCACTCTGCCTGGGTCT	NM_001244397.1	170
*IL1B for* *IL1B rev*	CCAAAGAGGGACATGGAGAA TTATATCTTGGCGGCCTTTG	XM_021085847.1	159
*IL6 for* *IL6 rev*	CACCAGGAACGAAAGAGAGC GTTTTGTCCGGAGAGGTGAA	NM_214399.1	193
*ITGAM for* *ITGAM rev*	AGAAGGAGACACCCAGAGCA GTAGGACAATGGGCGTCACT	XM_021086380.1	169
*NOS2 for* *NOS2 rev*	TGTTCAGCTGTGCCTTCAAC CAGAACTGGGGGTACATGCT	NM_001143690.1	175
*OPNMW for* *OPNMW rev*	GGGGAGCATCTTCACCTACA GATGATGGTCTCTGCCAGGT	NM_001011506.1	244
*RHO for* *RHO rev*	TCCAGGTACATCCCAGAAGG GCTGCCCATAGCAGAAGAAG	NM_214221.1	151
*RLP4 for* *RLP4 rev*	CAAGAGTAACTACAACCTTC GAACTCTACGATGAATCTTC	XM_005659862.3	164
*TNF for* *TNF rev*	CCACCAACGTTTTCCTCACT CCAAAATAGACCTGCCCAGA	JF831365.1	296

## Data Availability

The datasets generated during and/or analyzed during the current study are available from the corresponding authors on reasonable request.

## Supplementary Materials

The following supporting information can be downloaded at: https://www.mdpi.com/article/10.3390/biom12070990/s1, Figure S1: Evaluation of active microglia; Table S1: List of defined background, lower and upper threshold values.
