# Correction: Wagner et al. Impact of Primary RPE Cells in a Porcine Organotypic Co-Cultivation Model. *Biomolecules* 2022, *12*, 990

**DOI:** 10.3390/biom16081139

**Published:** 2026-08-05

**Authors:** Natalie Wagner, Armin Safaei, José Hurst, Pia A. Vogt, H. Burkhard Dick, Stephanie C. Joachim, Sven Schnichels

**Affiliations:** 1 Experimental Eye Research Institute, University Eye Hospital, Ruhr-University Bochum, 44892 Bochum, Germany; natalie.wagner@rub.de (N.W.); armin.safaei@rub.de (A.S.); pia.vogt@rub.de (P.A.V.); burkhard.dick@kk-bochum.de (H.B.D.); 2 Centre for Ophthalmology, University Eye Hospital Tübingen, 72076 Tübingen, Germany; jose.hurst@med.uni-tuebingen.de

## Error in Figure

In the original publication [1], it was discovered that some of the representative images provided in Figure 4 overlap with those published in another paper [2] and are incorrect. This resulted from an unintended mix-up and inadvertent reuse of image panels during the parallel preparation of two closely related studies. This occurred because the figures were prepared using a similar layout and organizational structure. The authors would like to replace Figure 4A of the following published paper [1]. The new, corrected version of Figure 4 appears below. The authors state that the scientific conclusions are unaffected. This correction was approved by the Academic Editor. The original publication has also been updated.

## Figures and Tables

**Figure 4:**
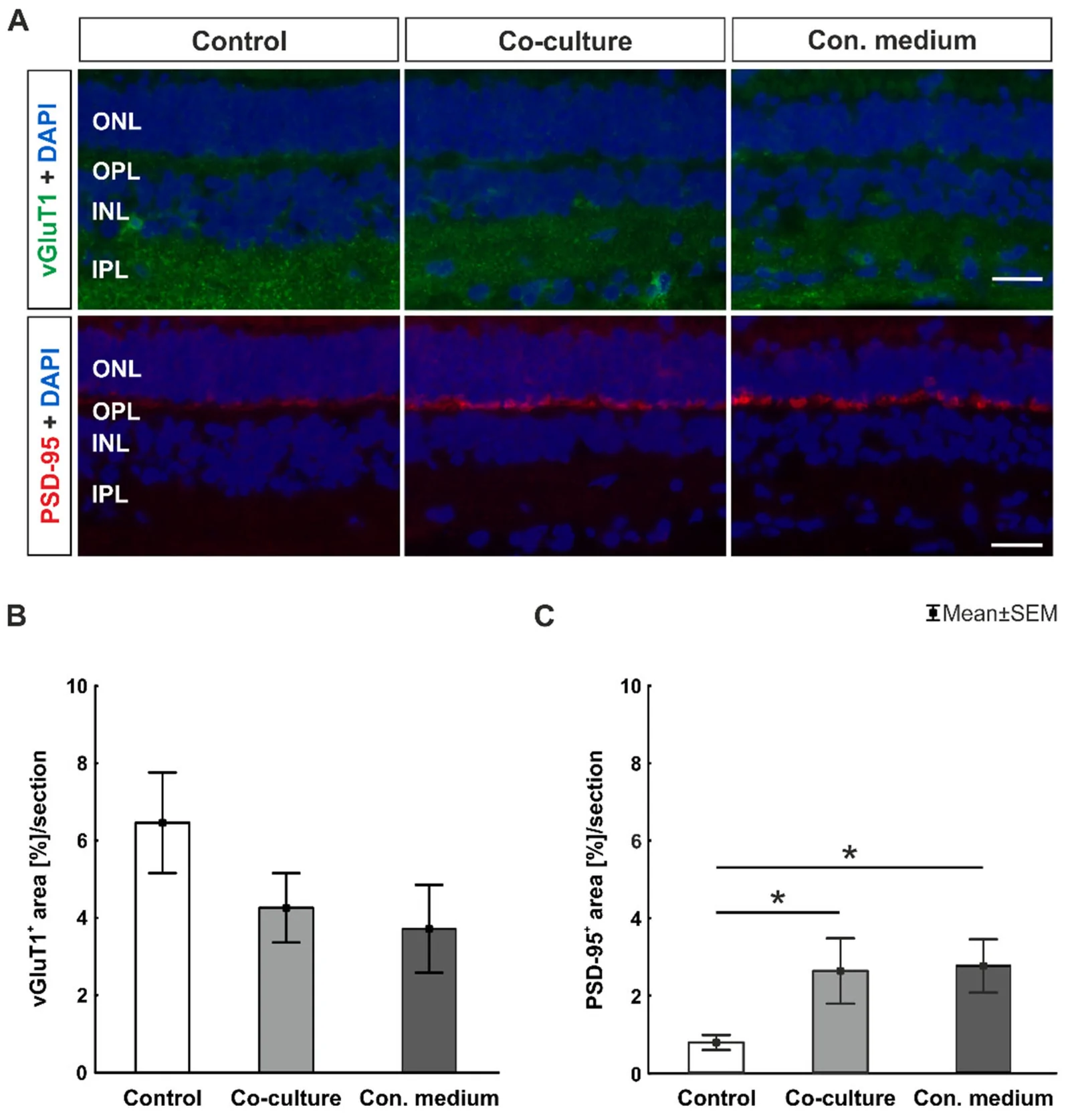
Unaltered synaptic density in cultivated retina. (**A**) To label the uptake of glutamate into synaptic vesicles at presynaptic nerve terminals, vGluT1 antibody was used (green). Whereas the PSD-95 antibody (red) stained the postsynaptic terminals. Cell nuclei were labeled with DAPI (blue). (**B**) vGluT1^+^ signal area was not altered in co-culture or conditioned medium groups compared to controls. (**C**) More PSD-95^+^ signal area was observed in the co-culture (*p* = 0.049) and conditioned medium group (*p* = 0.022) compared to controls. ONL, outer nuclear layer; OPL, outer plexiform layer; INL, inner nuclear layer; IPL, inner plexiform layer. Scale bar: 20 μm; values are presented as mean ± SEM, n = 8/group, * *p* < 0.05.
