# Supplementary material for: Impact of Primary RPE Cells in a Porcine Organotypic Co-Cultivation Model
Source: Biomolecules. 2022 Jul 16;12(7):990. doi: 10.3390/biom12070990 (PMC9313304; doi:10.3390/biom12070990)
Supplement: Supplementary file 1 [file biomolecules-12-00990-s001.zip › biomolecules-1716435-supplementary.pdf]

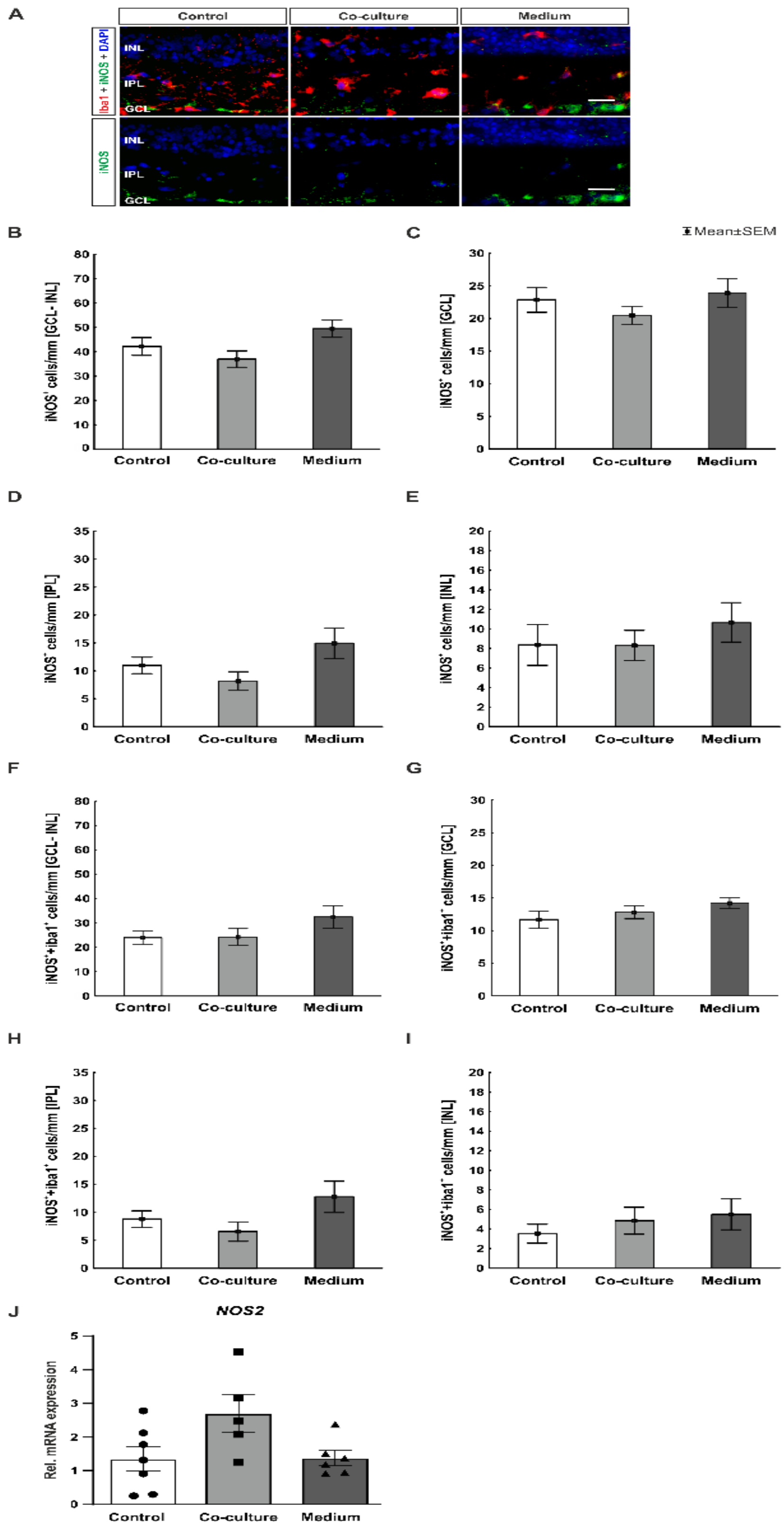

Figure S1. Evaluation of active microglia. (**A**) iNOS+ cells were stained (green). Active microglia were identified by the co-localization of iNOS and Iba1 (red), while cell nuclei were labelled with DAPI (blue). (**B**) The number of iNOS+ cells was similar in the GCL to INL. iNOS+ cell counts were also comparable in the GCL (**C**), IPL (**D**), and INL (**E**). (**F**) The number of iNOS+ and Iba1+ cells was comparable in the GCL to INL in all groups. Again, no significant differences in the number of iNOS+ and Iba1+ cells were detected in the

individual retinal layers, GCL (**G**), IPL (**H**), and INL (**I**). (**J**) The relative mRNA expression level of *NOS2* was not altered in all groups. INL, inner nuclear layer; IPL, inner plexiform layer; GCL, ganglion cell layer. Scale bar: 20 µm, values are presented as mean±SEM, (**A-I**) n=8/group, (**J**) n=6/group.

Table S1. List of defined background, lower and upper threshold values.

Staining	Background substraction	Lower threshold	Upper threshold
PSD-95	50	8.61	77.00
Rhodopsin	50	13.32	121.00
vGluT1	50	5.69	255.00
